# Fear response-based prediction for stress susceptibility to PTSD-like phenotypes

**DOI:** 10.1186/s13041-020-00667-5

**Published:** 2020-10-07

**Authors:** Min-Jae Jeong, Changhee Lee, Kibong Sung, Jung Hoon Jung, Jung Hyun Pyo, Joung-Hun Kim

**Affiliations:** 1 Department of Life Sciences, Pohang University of Science and Technology (POSTECH), Pohang, Gyungbuk 37673 Republic of Korea; 2 Department of Mathematics, Pohang University of Science and Technology (POSTECH), Pohang, Gyungbuk 37673 Republic of Korea; 3 Program in Neurosciences and Mental Health, Hospital for Sick Children, Toronto, Ontario Canada

**Keywords:** PTSD, Stress, Fear conditioning, ITI

## Abstract

Most individuals undergo traumatic stresses at some points in their life, but only a small proportion develop stress-related disorders such as anxiety diseases and posttraumatic stress disorder (PTSD). Although stress susceptibility is one determinant of mental disorders, the underlying mechanisms and functional implication remain unclear yet. We found that an increased amount of freezing that animals exhibited in the intertrial interval (ITI) of a stress-enhanced fear learning paradigm, predicts ensuing PTSD-like symptoms whereas resilient mice show ITI freezing comparable to that of unstressed mice. To examine the behavioral features, we developed a systematic analytical approach for ITI freezing and stress susceptibility. Thus, we provide a behavioral parameter for prognosis to stress susceptibility of individuals in the development of PTSD-like symptoms as well as a new mathematical means to scrutinize freezing behavior.

## Introduction

Posttraumatic stress disorder (PTSD) is a mental disorder triggered by exposure to traumatic stresses. PTSD is distinguished from other stress-induced disorders, including depression, schizophrenia, and general anxiety disorder, and thus was separately listed in the 5th edition of the Diagnostic and Statistical Manual (DSM-5) [1–3]. A characteristic symptom of PTSD is persistent re-experiencing or dreaming of traumatic episode(s), and the patients also exhibit fear generalization, exemplified by hypervigilance and exaggerated responses toward potential threats and even irrelevant cues [3, 4]. Although most people experience traumatic episodes at some points in their life, individual differences in stress susceptibility limit the development of PTSD symptoms to a minor faction (7–30% of the population) [5–7].

To obtain etiological and molecular insights into PTSD, several animal models have been developed, which recapitulate major PTSD symptoms, such as trigger-induced persistent and exaggerated learned fear and extinction resistance [7–11]. Outbred mice have normally been used to assess and compare the stress susceptibility of individual animals [8]. One criteria used to assess stress susceptibility is anxiety, despite the revision of the criteria for PTSD in the DSM-5 [12–14]. The stress-enhanced fear learning (SEFL) paradigm, with exposure to brief stresses rather than chronic stress, has been used to help distinguish trauma-related disorders from anxiety disorders [14–16]. However, a large number of behavioral tests are required to firmly verify whether each animal is susceptible or resilient to stressors, which are at risk of involving complications from various genetic factors for different behaviors [17, 18].

One of major PTSD-like symptoms is fear generalization, which can be measured as the ratio of freezing behavior toward a novel cue relative to that for a conditioned stimulus [10, 11, 19]. By employment of a modified SEFL model, we assessed fear generalization and fear recall after memory extinction to determine the stress susceptibility of individual animals through a new analytical algorithm. Our quantitative analyses revealed that stress susceptibility highly concurs with and is predicted by the freezing responses that subject animals showed in the intertrial interval (ITI) during fear conditioning. Furthermore, the ITI freezing responses can forecast the occurrence of PTSD-like behaviors, which substantiates the causal involvement of stress susceptibility in the development of PTSD-like symptoms. Altogether, the ITI freezing responses can serve as a predictive parameter for individual susceptibility and as a result, make a new prognostic means for future development of PTSD-like symptoms.

## Materials and methods

### Subject animals

Male C57BL/6 J mice were housed under a 12-h light/dark cycle with ad libitum access to food and water. All procedures for animal experiments were approved by the ethical review committee of POSTECH (Pohang University of Science & Technology), Korea, and performed in accordance with the relevant guidelines.

### Stress exposure

We turned to a modified behavioral protocol for acute traumatic stress, which had been originally developed for rats [20]. In brief, the used stressor was a 1-h restraint stress (immobilization in a ventilated Plexiglas tube) along with 60 inescapable tail shocks (1 mA, 1 s) delivered at pseudorandom intervals of 30 to 90 s with a shock generator (SCITECH, South Korea).

The elevated plus maze (EPM) was used to measure anxiety levels 7 days after traumatic stress exposure. The maze composed of 4 perpendicular arms (50 cm in length, 10 cm in width) was raised 60 cm above the floor and. Two arms had black 30 cm-high walls, whereas the other two arms had no walls. Mice were placed in the center of the EPM, facing an open arm, and were allowed to explore the maze for 15 min. A video camera was placed directly above the maze to monitor mouse movement.

### Fear conditioning paradigm

One week after the stress exposure, mice underwent habituation for 5 min for 2 consecutive days in context A, which was one of two identical chambers (17.75 cm × 17.75 cm × 30.5 cm) constructed of aluminum and Plexiglas walls (Coulbourn Instruments, Holliston, MA) with metal stainless steel rod flooring that was attached to a shock generator (model H13–15; Coulbourn Instruments). A sound cue for the conditioned stimulus (CS) was generated by a digital amplifier (EH2020; Elechorn, South Korea). Fear generalization, extinction, and retrieval after extinction training were carried out in modified versions of the context. Smooth black plastic flooring and walls, aspen bedding, a mild peppermint scent, and a single house light were used as context B for fear generalization. For fear extinction, smooth white plastic flooring and walls, corncob bedding, 1% acetate scent, and a single house light were used as context C. Mice were videotaped with an infrared digital camera, mounted on top of each chamber, for subsequent behavioral analyses. The contexts were thoroughly cleaned between sessions with alcohol for habituation and fear conditioning sessions and with distilled water for fear generalization, fear extinction, and fear recall after extinction training.

24 h after the second habituation period, fear conditioning was conducted in context A. After an initial 2 min acclimation period, mice were presented with 4 CS-unconditioned stimulus (US) pairings with a 90 s-average pseudorandom ITI (range 60–120 s; Supplemental Fig. 1A). The CS was a 10 kHz, 30 s, 80 dB tone, and the US was a 0.5 s, 0.4 mA foot shock which was co-terminated with CS. 60 s after the last pairing, mice were returned to their home cages. Fear generalization test was conducted 24 h later in context B with no habituation. After an initial 3 min of acclimation to context B, mice were exposed to 3 presentations of a novel cue (2 kHz, 30 s, 80 dB tone) with a 90 s ITI. These were followed by 3 presentations of the CS (10 kHz tone, 30 s, 80 dB tone) with the same ITI. 24 h later, mice underwent fear extinction in context C. After 2 min of acclimation to context C, there were 30 presentations of the CS with a 5 s ITI. Testing of extinction memory was conducted 24 h later, in which mice were returned to context C, with three presentations of the CS (90 s ITI) 2 min after the start of the session.

### Behavioral analyses

Freezing behavior was assessed with FreezeFrame software (Coulbourn) using video recordings throughout all sessions. Freezing was defined as the absence of movement (except respiration) for more than 1 s. Freezing duration was converted into a percentage score (*fz*) for the entire experiment. The freezing level was measured every 10 s except during the extinction session, for which freezing was measured every 5 s (as the ITI was shorter than 10 s). Freezing data were analyzed relative to the cue presentation timing.

The generalization index was defined as the ratio of average freezing elicited by a novel cue to that triggered by the CS in the fear generalization session. For individual animals, the generalization index was defined as $\sum \limits_{i=1}^{N_{\mathrm{trial}}}\left(\frac{fz_i^{\mathrm{novel}}}{fz_i^{\mathrm{CS}}}\right)$, where ${fz}_i^{\mathrm{novel}}$ and ${fz}_i^{\mathrm{CS}}$ are the percentages of freezing for the *i*^th^ tone trial in the testing session, and *N*_trial_ is the total number of trials [10].

### Modeling criteria for ITI freezing

ITI freezing data of susceptible and resilient mice were assumed to follow a normal distribution, *N*_resilient_ = *N*(μ_resilient_, σ_resilient_) and *N*_susceptible_ = *N*(μ_susceptible_, σ_susceptible_), where μ_resilient_ < μ_susceptible_ (see Supplemental Fig. 1). To determine which group a given test data α belongs to, two probabilities were compared: P_1_ = P(*N*_resilient_ < α) and P_2_ = P(*N*_susceptible_ > α). If P_1_ < P_2_, α belongs to the resilient group, and if P_1_ > P_2_, α belongs to the susceptible group. Thus, we defined the classification score function S as follows:

1 $$ \mathrm{S}\left(\upalpha \right)={\mathrm{P}}_1-{\mathrm{P}}_2=\mathrm{P}\ \left({N}_{\mathrm{resilient}}<\upalpha \right)+\mathrm{P}\left({N}_{\mathrm{susceptible}}<\upalpha \right) $$

If the score function of α is positive [S(α) > 0], then α belongs to the susceptible group; if S(α) is < 0, then α belongs to the resilient group.

### Data analysis

For *K*-means clustering of generalization indices and freezing levels, MATLAB was used with the following parameters: function, kmeans; distance, cityblock; replicates, 3000; options, opts. Receiver operating characteristic (ROC) curves were made for susceptible and resilient groups to evaluate the efficacy of our prediction method relative to *K*-means clustering.

Statistical analysis was performed using SPSS and GraphPad Prism 8. For correlation tests, the Pearson correlation test was used. *R* values are indicated in the legends of figures (see Supplemental Fig. 2). A Student’s unpaired *t* test or nonparametric Mann–Whitney *U* test was used to compare two independent groups. For multiple comparisons, one-way analysis of variance (ANOVA) or two-way repeated measures ANOVA with Tukey’s post hoc tests were utilized. All data are expressed as the means ± standard errors of the means (SEMs). *P* values of < 0.05 were considered statistically significant.

## Results

### Behavioral consequences of exposure to traumatic stressors

**Fig. 1:**
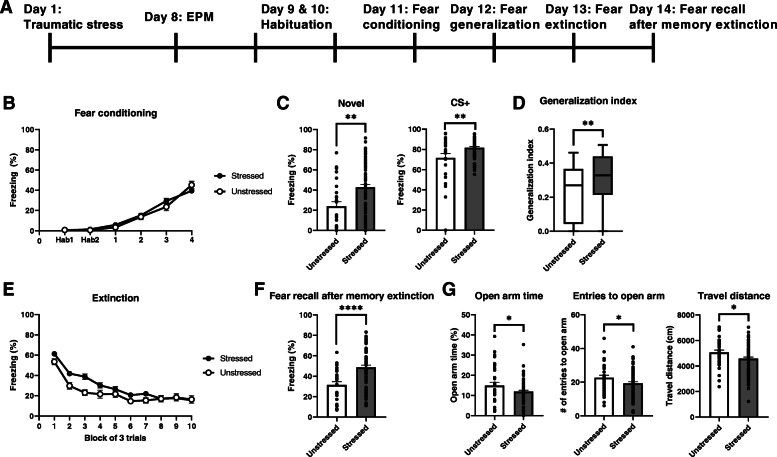
Acute traumatic stress increases anxious behaviors and fear responses. **a** A schematic of the behavioral test. Restraint and tail shock stresses are used as the traumatic stressor. 7 days later, anxiety levels are examined with an EPM test. Fear conditioning, fear generalization, fear extinction, and fear recall after memory extinction are then assessed sequentially. **b** Freezing responses of unstressed control (*n* = 26) and stressed (*n* = 79) mice during fear conditioning (two-way repeated measures ANOVA). **c-d** Fear generalization test. **c** Stressed mice show significantly enhanced freezing responses to a novel cue (left) and a CS (right) compared with those of unstressed control mice. **d** Generalization indices, calculated as the ratios of freezing responses to the novel cue and to the CS, are significantly higher in stressed mice than in control mice. **P < 0.01 (unpaired *t* test). **e** Freezing responses during extinction training are comparable between the two groups (two way repeated measures ANOVA). **f** Stressed mice show significantly increased freezing after extinction training. ****P < 0.0001 (unpaired t test). **g** EPM data from stressed and unstressed control mice. Stressed mice spend significantly less time in the open arms than control mice (left). Stressed mice make significantly fewer entries into open arms than controls (middle). Travel distances significantly differ between two groups (right). *P < 0.05; ns, not significant (unpaired t tests). Plots show means ± SEMs

We adopted a modified SEFL paradigm that combines a prior exposure to stress with auditory fear conditioning since this paradigm results in extinction resistance and models persistent re-experiencing of traumatic memories [9, 16]. When electric shocks were first applied during restraint stress as the traumatic event, additional electric shocks during fear conditioning acted as reminders of the traumatic stress. Then, the animals were tested for PTSD-like phenotypes such as fear generalization and fear recall after extinction procedures (Fig. 1a). In addition, the EPM was used to measure the anxiety levels of the mice.

Consistent with previous observation [7, 21–24], the acute traumatic stress did not affect fear conditioning or extinction learning, leading to comparable freezing responses between stressed and unstressed mice (Fig. 1b and e). The stressed mice displayed enhanced freezing responses to both CS and novel cues (Fig. 1c). Interestingly, they exhibited generalized responses to cues and impaired retrieval of extinction memory compared to the responses of the unstressed mice (Fig. 1d and f). The traumatic stress also tended to increase anxiety levels (Fig. 1g), as stressed animals had fewer entries to the open arms, spent less time there, and displayed less mobility than control unstressed mice (Fig. 1g). However, those parameters for anxiety levels did not show any apparent correlation with generalization indices or fear recall after memory extinction in both unstressed and stressed mice (Supplemental Fig. 2), suggesting that stress-induced alteration of anxiety levels is indifferent to fear modulation per se, while traumatic stresses are likely to affect fear responses and anxiety levels.

### Animal classification with PTSD-like phenotypes

**Fig. 2:**
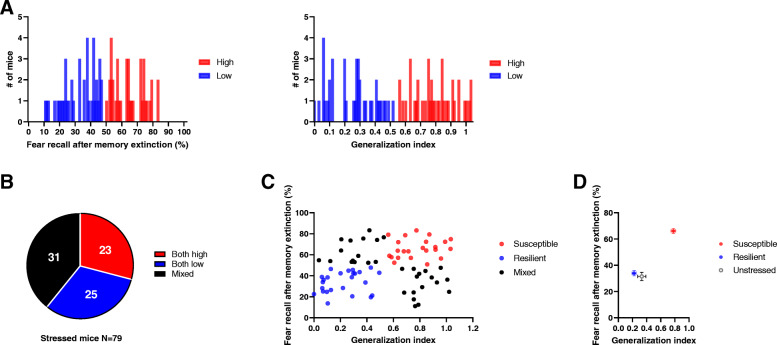
Classification of stressed mice through indices of fear generalization and extinction resistance. **a** *K*-means clustering analysis of freezing responses that stressed mice (*n* = 79) display one day after extinction training. Red, high, 38 mice; blue, low, 41 mice (left). *K*-means clustering analysis of generalization indices of stressed mice (*n* = 79). Red, high, 39 mice; blue, low, 40 mice (right). **b** Distribution of mice clustered and denoted as “both high” from fear recall and generalization indices (*n* = 23 mice [29.11%]), “both low” from both (*n* = 25 mice [31.65%]), and “mixed” for opposite results from two indices (*n* = 31 mice [39.24%]). **c** *K*-means clustering analysis using fear recall and generalization indices show distinct distributions of susceptible mice (red, both high), resilient mice (blue, both low), and nonallocated mice (black, mixed). **d** Distributions of fear recall and generalization indices for susceptible, resilient, and unstressed control mice (means ± SEMs)

Fear generalization and impairments in extinction memory typically represent PTSD-like symptoms [23–25]. Initially, we attempted to categorize the stressed mice exhibiting generalization and extinction resistance via *K*-means clustering, an unsupervised learning algorithm with a vector quantization method (Fig. 2a). This clustering analysis revealed 3 groups of animals: animals showing higher indices for both assessments, regarded as susceptible (*n =* 23 mice [29.11%]); animals showing lower indices for both, regarded as resilient (*n* = 25 mice [31.65%]); and animals showing mixed indices, denoted as mixed (*n* = 31 [39.24%]) (Fig. 2b and c). Interestingly, 26 unstressed control mice had means and distributions of two parameters comparable to those of the resilient group of stressed mice but not those of the susceptible group (Fig. 2d).

**Fig. 3:**
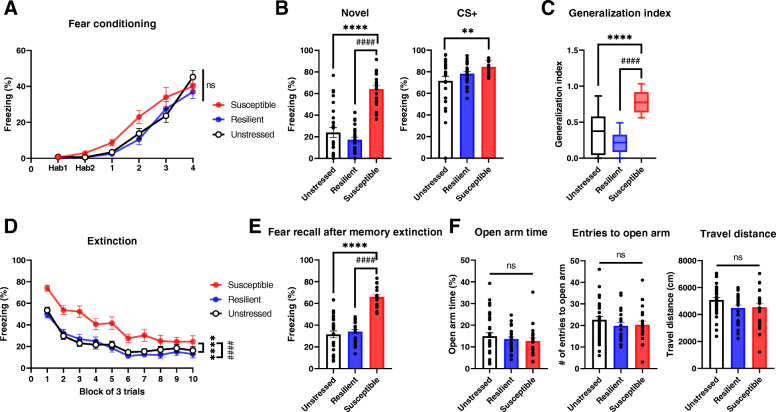
Similar anxiety levels but increased fear responses from susceptible mice. **a** Freezing responses to CS during fear conditioning are similar among groups (two-way repeated measures ANOVA). **b-c** Susceptible mice exhibit enhanced fear generalization. **b** Susceptible mice show increased freezing responses to the novel cue compared with those of resilient and unstressed mice (left) and increased freezing responses to the CS compared with those of unstressed mice but not resilient ones (right). **c** Generalization indices of susceptible mice are significantly higher than those of the other groups. **P < 0.01, ****P < 0.0001, ^####^*P* < 0.0001 (one-way ANOVAs). **d** Fear extinction is significantly impaired in susceptible mice. ****P < 0.0001, ^####^*P* < 0.0001 (two-way repeated measures ANOVA, Tukey’s test). **e** Susceptible mice exhibit increased freezing responses 24 h after memory extinction compared with those of other groups. ****P < 0.0001, ^####^*P* < 0.0001 (one-way ANOVA). **f** All groups exhibit comparable anxious behaviors in the EPM for times spent in open arms (left), numbers of entries into open arms (middle), and the travel distance (right) (one-way ANOVAs). Plots show means ± SEMs

Stressed animals, regardless of being either susceptible or resilient, and unstressed controls exhibited similar learning curves during fear conditioning (Fig. 3a). Notably, susceptible mice showed higher freezing responses to novel cues and generalization indices than resilient and control mice while the fear responses to CS was comparable between susceptible and resilient mice (Fig. 3b and c). Moreover, fear extinction training and the retrieval of extinction memory were significantly impaired in the susceptible group (Fig. 3d and e). Interestingly, anxiety behaviors were similar among all the groups (Fig. 3f), indicating that anxiety levels were not altered by susceptibility traits exhibited by mice after stress exposure.

### Increases in ITI freezing responses by susceptible mice

**Fig. 4:**
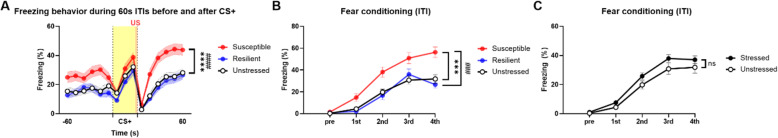
Stress exposure results in increased freezing in the ITI during fear conditioning only in susceptible mice. **a** Susceptible mice exhibit enhanced freezing 60 s before and 60 s after CS presentation compared with that by unstressed and resilient mice. Those data are mean values of freezing responses from 4 trials of 60 s before and 60 s after CS-US pairing. US was presented and co-terminated with CS. ****P < 0.0001, ^####^*P* < 0.0001 (two-way repeated measures ANOVA, Tukey’s test). **b** Susceptible mice exhibit increased freezing responses compared with those by unstressed and resilient mice in the ITI. ***P < 0.001, ^###^*P* < 0.001 (two-way repeated measures ANOVA, Tukey’s test). **c** Stressed mice exhibit comparable freezing responses in ITI to those by unstressed control mice. ns, not significant (two-way repeated measures ANOVA). Plots show means ± SEMs

Because anxiety levels and fear learning in susceptible mice were not different from those of other groups (Fig. 3a and f), we sought to identify which behavioral features during fear conditioning could define or forecast the susceptibility traits observed after fear conditioning, i.e., in generalization and extinction resistance. A close examination of freezing responses indicated that susceptible mice spent more time freezing in the 60 s before and after CS presentation than resilient and unstressed control mice (Fig. 4a). We also observed an increase in freezing in the ITI by the susceptible animals (Fig. 4b). However, the differential freezing responses were masked between unstressed and stressed mice when resilient and susceptible mice were combined into one stressed group (Fig. 4C).

### Prediction model with ITI freezing for susceptibility traits

Given the strong association between ITI freezing and fear generalization/extinction resistance, we attempted to construct a model whereby we could predict the susceptibility of animals to PTSD-like symptoms by using the ITI freezing data. To this end, we set distribution areas for susceptible and resilient groups using the means and standard deviations of ITI freezing responses at each time point. Then, we calculated a classification score from ITI freezing data for each mouse (see Materials and Methods).

**Fig. 5:**
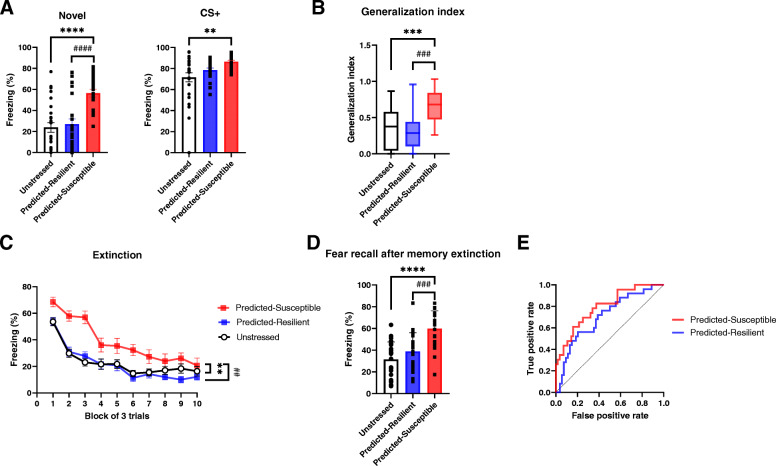
Validation of the prediction model for stress susceptibility to PTSD-like behaviors. **a-b** Freezing responses during the fear generalization test. **a** Predicted-susceptible mice show increased freezing to a novel cue compared with that by unstressed control and predicted resilient mice (left). Predicted-susceptible mice show increased freezing responses to the CS compared with those by control mice but not predicted-resilient ones (right). **b** Predicted-susceptible mice show increases in generalization indices compared with those for control and predicted-resilient mice. **P < 0.01, ***P < 0.001, ****P < 0.0001, ^###^*P* < 0.001, ^####^*P* < 9 0.0001 (one-way ANOVA, Tukey’s test). **c** Predicted-susceptible mice show increased freezing responses during extinction training compared with those by unstressed control and predicted-resilient mice. **P < 0.01, ^##^*P* < 0.01 (two-way repeated measures ANOVA, Tukey’s test). **d** Predicted-susceptible mice exhibit enhanced freezing responses 24 h after extinction training compared with those by unstressed control and predicted resilient mice. ****P < 15 0.0001, ^###^*P* < 0.001 (one-way ANOVA, Tukey’s test). **e** ROC curves for predicted-susceptible and predicted-16 resilient mice. The AUC for the predicted-susceptible group is 0.7950 (95% confidence interval, 0.6884–0.9017; ****P < 0.0001) and that for predicted-resilient group is 0.7067 (95% confidence interval, 0.5845–0.8288; **P < 0.01)

We reclassified 79 stressed mice using our prediction modeling criteria. According to the disease rate of PTSD in a human study [5], we also designated animals with classification scores in the top 30% as susceptible and those with scores in the bottom 30% as resilient. The susceptible group, categorized using prediction criteria for ITI freezing data, exhibited increased freezing responses to novel cues, enhanced generalization indices, and extinction resistance, while the animals displayed the similar freezing response to CS during generalization test compared with the predicted-resilient mice (Fig. 5a-d). Note that a similar pattern of results was also observed in Fig. 3b. We also used ROC curves for the susceptible and resilient groups (Fig. 5e) to further validate the efficacy of our prediction model. The areas under the curves (AUCs) for the predicted susceptible and resilient groups were 0.7950 and 0.7067, respectively, which indicated that the AUCs differed significantly from the random discrimination level (*P* < 0.0001 and *P* < 0.01, respectively). Altogether, these data substantiated that our prediction method was reliable and sufficient to predict the stress susceptibility to PTSD-like phenotypes [26, 27].

## Discussion

We investigated whether an exposure to a traumatic stress results in specific behavioral alterations during fear conditioning in mice susceptible to PTSD-like phenotypes. This study provides several important insights into stress susceptibility: (1) acute traumatic stress results in anxious behaviors and enhanced fear responses; (2) stress-induced anxious behaviors are not coupled to altered fear responses; and (3) freezing in the ITI during fear conditioning predicts stress susceptibility to PTSD-like phenotypes.

As individuals with PTSD often suffer from comorbid mood and anxiety disorders [3], further revisions for separate PTSD diagnoses are suggested for the next DSM [28]. Furthermore, it remains inconclusive whether anxiety tests are an appropriate measure for PTSD [29]. While fear and anxiety share certain neuronal components and modules for their establishment and regulation, they rely on separate neural circuits and mechanisms [30]. Accordingly, the anxiety and fear symptoms in PTSD patients arise differentially and are independently controlled [31, 32]. Although traumatic stress can induce both anxious behaviors and enhanced fear responses, we did not observe any significant correlation between stress-induced anxious behaviors and PTSD-like phenotypes, such as fear generalization and neither extinction resistance (Supplemental Fig. 2). This observation suggests that stress-induced anxious behaviors are not a prerequisite for the manifestation of stress-induced PTSD-like phenotypes, whereas these parallel behaviors interact and modulate each other.

24 h after fear conditioning, resilient and susceptible mice revealed no difference in the responses to the CS during the fear generalization test (Figs. 3b and 5a). This finding indicates that susceptible and resilient mice have comparable fear memory formation and retrieval. Despite the similarity, however, resilient mice exhibited significantly reduced fear generalization with decreased freezing during fear extinction and fear recall after memory extinction. Taken together, these data support the idea that the reduced PTSD phenomena in resilient mice specifically results from their capability to overcome the effects of traumatic stresses, but would not be attributable to differences in how the stress was experienced, how the stress memory was formed, or in the ability to retrieve the stress memory [33, 34].

We propose a new analysis algorithm in which freezing data taken from the ITI during fear conditioning can be used to predict the stress susceptibility of subject animals to PTSD-like phenotypes. In fact, the ITI may play critical roles for several types of memories [35–37]. For instance, the duration of the ITI in the training procedure is inversely related to short-term memory recall [38]. A shorter ITI improves the learning performance of autistic children [39], but a longer ITI promotes a better performance for Pavlovian feature discriminations [40]. The ITI duration may also intervene in memory extinction, as subjects who received variable ITIs reinstated fear memory better than those receiving a fixed ITI [37]. Despite the potential importance of the ITI, only behavioral features of the conditioned/unconditioned responses to stimuli have been examined, while those that occur during the ITIs have been largely ignored thus far. This is likely due to the lack of predictive attributes of ITI freezing displayed by unstressed animals. Here, our behavioral data indicate that ITI freezing is a valuable and prognostic parameter for stress susceptibility of animals exposed to traumatic stress.

The present studies highlight a potentially important role of ITI freezing by stressed mice in predicting their stress susceptibility. While it is unknown how ITI freezing represents stress susceptibility to PTSD-like phenomena, epigenetic processes such as DNA methylation, histone modification, and microRNAs may be involved, as previously surmised [41, 42]. Mechanisms by which ITI freezing defines stress susceptibility to PTSD merit further investigation.

## Supplementary information

**Additional file 1: Supplemental Fig. 1.** ITI freezing as a criterion for prediction of stress susceptibility. (A) A behavioral timeline. After an initial 120 s acclimation period, mice were subjected to 4 trials of tone CS. CS were co-terminated with a foot shock. Each CS lasted for 30 s, and was presented in pseudorandom order with a 90 s ITI (range 60–120 s). (B) Distributions of ITI freezing data from susceptible (red) and resilient (blue) mice. Distributions of freezing data during the 1st ITI (top left): resilient, μ = 2.1403, σ = 3.587; susceptible, μ = 13.3774, σ = 16.6243; green, 4.4324 where normalized Z1 = Z2. Distributions of freezing data during the 2nd ITI (top right): resilient, μ = 16.7149, σ = 18.3964; susceptible, = 35.7977, σ = 21.6703; green, 25.606, where normalized Z1 = Z2. Distributions of freezing data during the 3th ITI (bottom left): resilient, μ = 36.0558, σ = 24.4761; susceptible, μ = 50.4523, σ = 24.1190; green, 43.1805, where normalized Z1 = Z2. Distributions of freezing data during the 4th ITI (bottom right): resilient, μ = 26.6585, σ = 16.1910; susceptible, μ = 54.9368, σ = 22.3665; green, 38.6686, where normalized Z1 = Z2. (**C**) Criterion for categorization of mice into susceptible and resilient groups. Black line is the criterion that connects the green points in panels B. Plots show means ± SEMs.

**Additional file 2: Supplemental Fig. 2.** Anxious behaviors do not correlate with occurrence of PTSD-like phenotypes. (A) Anxiety levels of stressed mice do not correlate with PTSD-like behaviors. Neither the amount of time that stressed mice spent in the open arms of the EPM (top left, Pearson correlations, *R* = − 0.03426) nor the number of entries that stressed mice made into open arms (bottom left, *R* = − 0.01298) correlated with the fear generalization indices. Neither the amount of time that stressed mice spent in the open arms of the EPM (top right, *R* = − 0.01298) nor the number of entries that stressed mice made into open arms (bottom right, *R* = 0.06744) correlated with freezing responses 24 h after memory extinction. (B) Anxiety levels of unstressed control mice do not correlate with PTSD-like behaviors. Neither the amount of time that control mice spent in the open arms of the EPM (top left, *R* = − 0.09436) nor the number of entries that control mice made into open arms (bottom left, *R* = 0.09593) correlated with the fear generalization indices. Neither the amount of time that control mice spent in the open arms of the EPM (top right, *R* = − 0.2067) nor the number of entries that stressed mice made into open arms (bottom right, *R* = 0.09174) correlated with freezing responses 24 h after memory extinction.

## Data Availability

All data generated or analyzed during this study are included in this published article.

## Abbreviations

PTSD: posttraumatic stress disorder
SEFL: stress-enhanced fear learning
DSM: Diagnostic and Statistical Manual
ITI: intertrial interval
CS: conditioned stimulus
US: unconditioned stimulus
EPM: elevated plus maze
ANOVA: analysis of variance
SEM: standard error of the mean
ROC: receiver operating characteristic
AUC: area under the curve
