# Supplementary material for: Fear response-based prediction for stress susceptibility to PTSD-like phenotypes
Source: Mol Brain. 2020 Oct 7;13:134. doi: 10.1186/s13041-020-00667-5 (PMC7539418; doi:10.1186/s13041-020-00667-5)
Supplement: Supplementary file 1 — Additional file 1: Supplemental Fig. 1. ITI freezing as a criterion for prediction of stress susceptibility. (A) A behavioral timeline. After an initial 120 s acclimation period, mice were subjected to 4 trials of tone CS. CS were co-terminated with a foot shock. Each CS lasted for 30 s, and was presented in pseudorandom order with a 90 s ITI (range 60–120 s). (B) Distributions of ITI freezing data from susceptible (red) and resilient (blue) mice. Distributions of freezing data during the 1st ITI (top left): resilient, μ = 2.1403, σ = 3.587; susceptible, μ = 13.3774, σ = 16.6243; green, 4.4324 where normalized Z1 = Z2. Distributions of freezing data during the 2nd ITI (top right): resilient, μ = 16.7149, σ = 18.3964; susceptible, = 35.7977, σ = 21.6703; green, 25.606, where normalized Z1 = Z2. Distributions of freezing data during the 3th ITI (bottom left): resilient, μ = 36.0558, σ = 24.4761; susceptible, μ = 50.4523, σ = 24.1190; green, 43.1805, where normalized Z1 = Z2. Distributions of freezing data during the 4th ITI (bottom right): resilient, μ = 26.6585, σ = 16.1910; susceptible, μ = 54.9368, σ = 22.3665; green, 38.6686, where normalized Z1 = Z2. (C) Criterion for categorization of mice into susceptible and resilient groups. Black line is the criterion that connects the green points in panels B. Plots show means ± SEMs. [file 13041_2020_667_MOESM1_ESM.pdf]

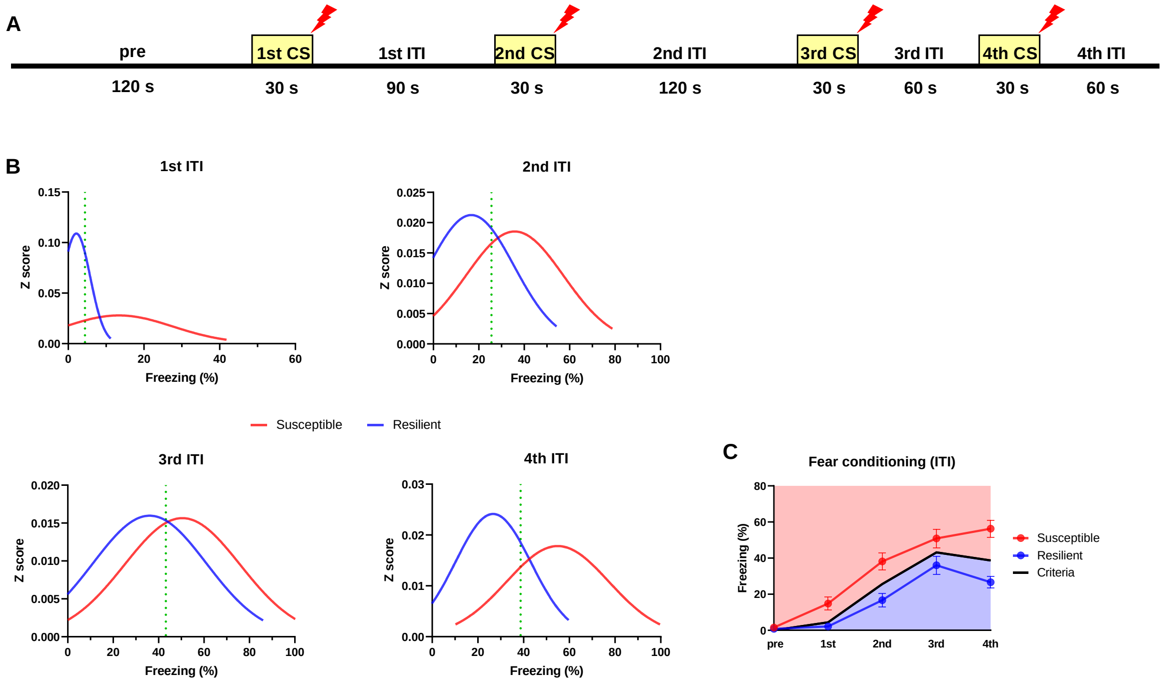

Supplemental Fig. 1 ITI freezing as a criterion for prediction of stress susceptibility.

(**A**) A behavioral timeline. After an initial 120 s acclimation period, mice were subjected to 4 trials of tone CS. CS were co-terminated with a foot shock. Each CS lasted for 30 s, and was presented in pseudorandom order with a 90 s ITI (range 60-120 s).

(**B**) Distributions of ITI freezing data from susceptible (red) and resilient (blue) mice. Distributions of freezing data during the 1st ITI (top left): resilient, $\mu = 2.1403$, $\sigma = 3.587$; susceptible, $\mu = 13.3774$, $\sigma = 16.6243$; green, 4.4324 where normalized $Z_1 = Z_2$. Distributions of freezing data during the 2nd ITI (top right): resilient, $\mu = 16.7149$, $\sigma = 18.3964$; susceptible, $\mu = 35.7977$, $\sigma = 21.6703$; green, 25.606, where normalized $Z_1 = Z_2$. Distributions of freezing data during the 3th ITI (bottom left): resilient, $\mu = 36.0558$, $\sigma = 24.4761$; susceptible, $\mu = 50.4523$, $\sigma = 24.1190$; green, 43.1805, where normalized $Z_1 = Z_2$. Distributions of freezing data during the 4th ITI (bottom right): resilient, $\mu = 26.6585$, $\sigma = 16.1910$; susceptible, $\mu = 54.9368$, $\sigma = 22.3665$; green, 38.6686, where normalized $Z_1 = Z_2$.

(**C**) Criterion for categorization of mice into susceptible and resilient groups. Black line is the

criterion that connects the green points in panels B.

Plots show means ± SEMs.
