# Supplementary material for: Fear response-based prediction for stress susceptibility to PTSD-like phenotypes
Source: Mol Brain. 2020 Oct 7;13:134. doi: 10.1186/s13041-020-00667-5 (PMC7539418; doi:10.1186/s13041-020-00667-5)
Supplement: Supplementary file 2 — Additional file 2: Supplemental Fig. 2. Anxious behaviors do not correlate with occurrence of PTSD-like phenotypes. (A) Anxiety levels of stressed mice do not correlate with PTSD-like behaviors. Neither the amount of time that stressed mice spent in the open arms of the EPM (top left, Pearson correlations, R = − 0.03426) nor the number of entries that stressed mice made into open arms (bottom left, R = − 0.01298) correlated with the fear generalization indices. Neither the amount of time that stressed mice spent in the open arms of the EPM (top right, R = − 0.01298) nor the number of entries that stressed mice made into open arms (bottom right, R = 0.06744) correlated with freezing responses 24 h after memory extinction. (B) Anxiety levels of unstressed control mice do not correlate with PTSD-like behaviors. Neither the amount of time that control mice spent in the open arms of the EPM (top left, R = − 0.09436) nor the number of entries that control mice made into open arms (bottom left, R = 0.09593) correlated with the fear generalization indices. Neither the amount of time that control mice spent in the open arms of the EPM (top right, R = − 0.2067) nor the number of entries that stressed mice made into open arms (bottom right, R = 0.09174) correlated with freezing responses 24 h after memory extinction. [file 13041_2020_667_MOESM2_ESM.pdf]

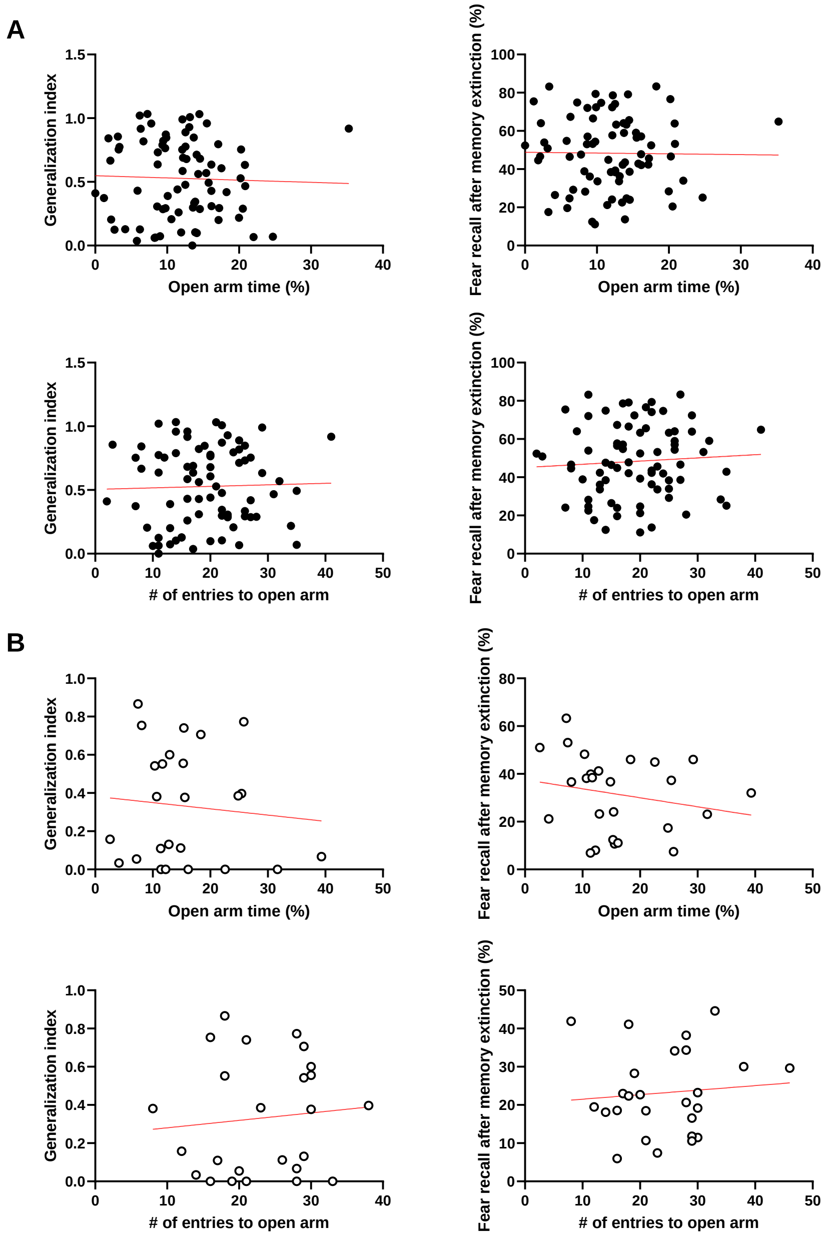

Supplemental Fig. 2 Anxious behaviors do not correlate with occurrence of PTSD-like phenotypes.

(**A**) Anxiety levels of stressed mice do not correlate with PTSD-like behaviors. Neither the amount of time that stressed mice spent in the open arms of the EPM (top left, Pearson correlations, R = -0.03426) nor the number of entries that stressed mice made into open arms (bottom left, R = -0.01298) correlated with the fear generalization indices. Neither the amount

of time that stressed mice spent in the open arms of the EPM (top right, R = -0.01298) nor the number of entries that stressed mice made into open arms (bottom right, R = 0.06744) correlated with freezing responses 24 h after memory extinction.

(**B**) Anxiety levels of unstressed control mice do not correlate with PTSD-like behaviors. Neither the amount of time that control mice spent in the open arms of the EPM (top left, R = -0.09436) nor the number of entries that control mice made into open arms (bottom left, R = 0.09593) correlated with the fear generalization indices. Neither the amount of time that control mice spent in the open arms of the EPM (top right, R = -0.2067) nor the number of entries that stressed mice made into open arms (bottom right, R = 0.09174) correlated with freezing responses 24 h after memory extinction.
